# Erratum to: Outcomes of patients with ST-segment myocardial infarction admitted during the COVID-19 pandemic: A prospective, observational study from a tertiary care center in Germany

**DOI:** 10.1007/s00059-022-05127-5

**Published:** 2022-07-19

**Authors:** M. Rattka, C. Winsauer, L. Stuhler, K. Thiessen, M. Baumhardt, T. Stephan, W. Rottbauer, A. Imhof

**Affiliations:** Department of Cardiology, Ulm University Medical Centre, Albert Einstein Allee 23, 89081 Ulm, Germany

**Erratum to:**

**Herz 2021**

10.1007/s00059-021-05058-7

Due to a type-setting error by the publisher, the licence information was missing from this article. It should read: “**Open Access**. This article is licensed under a Creative Commons Attribution 4.0 International License, which permits use, sharing, adaptation, distribution and reproduction in any medium or format, as long as you give appropriate credit to the original author(s) and the source, provide a link to the Creative Commons licence, and indicate if changes were made. The images or other third party material in this article are included in the article’s Creative Commons licence, unless indicated otherwise in a credit line to the material. If material is not included in the article’s Creative Commons licence and your intended use is not permitted by statutory regulation or exceeds the permitted use, you will need to obtain permission directly from the copyright holder. To view a copy of this licence, visit http://creativecommons.org/licenses/by/4.0/.”

The original article has been corrected.

